# POSITIVE PSYCHOLOGICAL DETERMINANTS OF DEMENTIA IN JAPAN

**DOI:** 10.1093/geroni/igz038.685

**Published:** 2019-11-08

**Authors:** Kokoro Shirai, Kokoro Shirai, Hiroyasu Iso, Ryoto Sakaniwa, Katsunori Kondo

**Affiliations:** 1 Graduate School of Medicine Osaka University, Suita, Osaka, Japan; 2 Graduate School of Medicine Osaka University, Osaka, Osaka, Japan; 3 Center for Preventive Medical Science, Chiba University, chiba, Chiba, Japan

## Abstract

We examined the effects of sustained levels of happiness on dementia incidence among Japanese elderly from the Japan Gerontological Evaluation Study. Data were from 12,051 community-dwelling participants aged 65 and over, free from dementia or other chronic conditions at baseline (2010). A survey was conducted in 31 administrative districts from Japan. Participants were followed-up for six years, and the incidence of dementia or death was assessed through National Public Long-Term Care Insurance and Resident Registry, Japan. We conducted survival analysis using the Cox proportional hazard model with a competing risk analysis accounting for death. The adjusted differences in days for dementia onset were estimated using Laplace regression models. In this analytical sample, we observed 520 men and 693 women with dementia and found that higher levels of sustained happiness were associated with reduced risks of onset of dementia among Japanese elderly. These findings suggest a protective role of psychological mood.

